# *N*-methyl d-aspartate receptor hypofunction reduces visual contextual integration

**DOI:** 10.1167/jov.21.6.9

**Published:** 2021-06-15

**Authors:** Alexander Schielke, Bart Krekelberg

**Affiliations:** 1 Center for Molecular and Behavioral Neuroscience, Rutgers University, Newark, NJ, USA; 2 Behavioral and Neural Sciences Graduate Program, Rutgers University, Newark, NJ, USA; 3 Center for Molecular and Behavioral Neuroscience, Rutgers University, Newark, NJ, USA

**Keywords:** surround suppression, gain control, eye movements, NMDA receptor, schizophrenia

## Abstract

Visual cognition is finely tuned to the elements in a scene but also relies on contextual integration to improve visual detection and discrimination. This integration is impaired in patients with schizophrenia. Studying impairments in contextual integration may lead to biomarkers of schizophrenia, tools to monitor disease progression, and, in animal models, insight into the underlying neural deficits. We developed a nonhuman primate model to test the hypothesis that hypofunction of the *N*-methyl d-aspartate receptor (NMDAR) impairs contextual integration.

Two male rhesus macaques (*Macaca mulatta*) were trained to indicate which of two patterns on the screen had the highest contrast. One of these patterns appeared in isolation, and the other was surrounded by a high-contrast pattern. In humans, this high-contrast context is known to lead to an underestimation of contrast. This so-called Chubb illusion is thought to result from surround suppression, a key contextual integration mechanism. To test the involvement of NMDAR in this process, we compared animals’ perceptual bias with and without intramuscular injections of a subanesthetic dose of the NMDAR antagonist ketamine.

In the absence of ketamine, the animals reported a Chubb illusion - matching reports in healthy humans. Hence, monkeys - just like humans - perform visual contextual integration. This reaffirms the importance of nonhuman primates to help understand visual cognition. Injection of ketamine significantly reduced the strength of the illusion and thus impaired contextual integration. This supports the hypothesis that NMDAR hypofunction plays a causal role in specific behavioral impairments observed in schizophrenia.

## Introduction

In current practice, visual abnormalities are not considered a core aspect of schizophrenia (Sz). Yet, two-thirds of patients report alterations to their everyday visual experience (Keane, Cruz, Paterno, & Silverstein, 2018), and more than a quarter experience visual hallucinations (Blom et al., 2014). Because the neural mechanisms of vision are relatively well understood, studying visual perceptual abnormalities in Sz offers an opportunity to understand the circuit-level causes of the disorder (Silverstein, Keane, Wang, Mikkilineni, Paterno, Papathomas, & Feigenson, 2013) and potentially develop psychometric diagnostic tests for Sz (Schiffman et al., 2006; Schubert, Henriksson, & McNeil, 2005).

**Figure 1.**
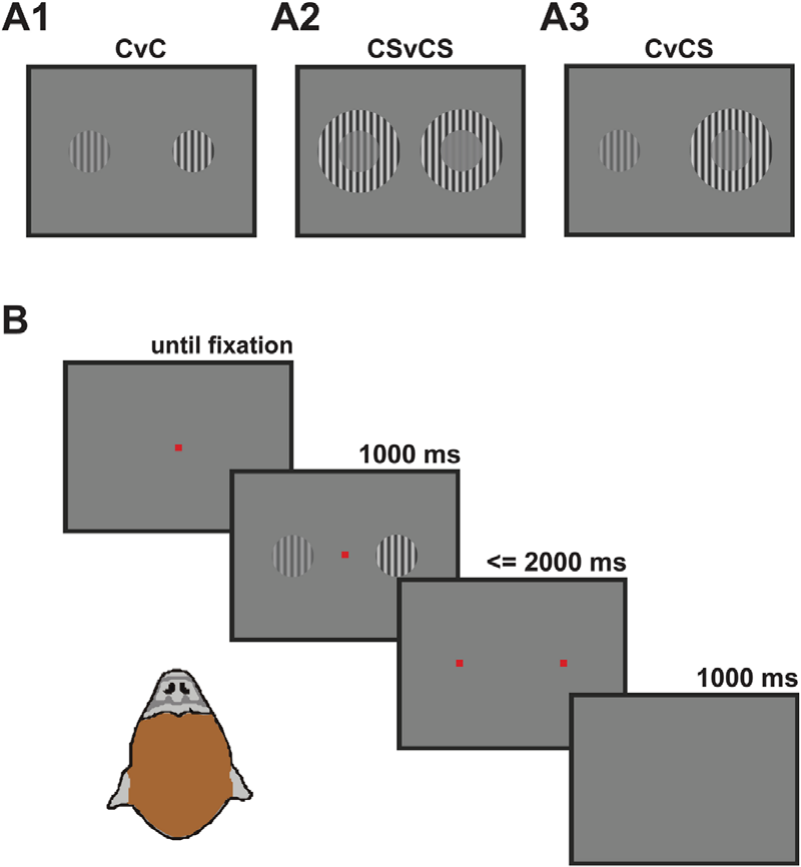
Experimental paradigm. (**A**) Example stimuli for the three conditions. The left center grating has the same contrast in each example. In A1 (CvC condition), the right grating has the higher contrast; the monkey would be rewarded for making an eye movement to the right red dot. In A2 (CSvCS condition), the higher contrast center grating is on the left. A3 (CvCS condition) shows an example of the Chubb illusion; both center gratings have the same physical contrast but to healthy human observers the left center grating appears to have higher contrast than the right center grating with the high-contrast surround. (**B**) Trial sequence. As soon as the monkey fixated the central red dot, two gratings appeared for 1 second, followed by two red dots. The monkey had 2 seconds to make an eye movement toward one of the red dots and thereby indicate which of the gratings had the higher contrast.

People living with Sz often describe visual scenes as isolated, fragmented elements (Uhlhaas & Mishara, 2007); this and several other visual abnormalities in Sz suggest a reduced ability to integrate visual elements across the scene. The psychometric tools of vision science allow careful quantification of these impairments as, for example, a reduced sensitivity to illusory contours of Kanisza shapes (Keane, Joseph, & Silverstein, 2014), as well as reduced biases in the Ebbinghaus illusion (Horton & Silverstein, 2011) and the Ponzo illusion (Kantrowitz, Butler, Schecter, Silipo, & Javitt, 2009). Here, we focus on the so-called Chubb illusion of perceived contrast, in which healthy human observers underestimate the contrast of a visual pattern surrounded by a high-contrast pattern. Figure 1 shows an example using the patterns (oriented gratings) we used in our study. For such oriented gratings, the illusion is sometimes called orientation-dependent surround suppression (ODSS); patients with Sz have substantially reduced ODSS (Dakin, Carlin, & Hemsley, 2005; Schallmo, Sponheim, & Olman, 2013; Schallmo, Sponheim, & Olman, 2015). The illusion likely results from center-surround interactions in the visual cortex (Jones, Grieve, Wang, & Sillito, 2001). Combining this with the role of *N*-methyl d-aspartate receptor (NMDAR) in recurrent connectivity (Self, Kooijmans, Supèr, Lamme, & Roelfsema, 2012) leads to the prediction that NMDAR hypofunction reduces susceptibility to the illusion.

Since the early 1990s, the view that NMDAR hypofunction plays an important role in the etiology of Sz has gained substantial support (Frohlich & Van Horn, 2014; Javitt, Zukin, Heresco-Levy, & Umbricht, 2012). In healthy volunteers (Krystal et al., 1994) and in patients (Lahti, Koffel, Laporte, & Tamminga, 1995), the administration of a subanesthetic dose of the NMDAR antagonist ketamine can induce hallucinations and other symptoms associated with Sz. Conversely, the NMDAR co-agonists glycine and d-serine reduce symptoms of Sz (Heresco-Levy, Javitt, Ermilov, Mordel, Horowitz, & Kelly, 1996; Javitt, Zylberman, Zukin, Heresco-Levy, & Lindenmayer, 1994; Tsai, Yang, Chung, Lange, & Coyle, 1998). The NMDAR hypofunction hypothesis appears most successful outside the frontostriatal “jurisdiction” of dopamine (Frohlich & Van Horn, 2014) in the sensory cortices. For example, the reduced responses to oddball stimuli (mismatch negativity) found in Sz can be recapitulated by the administration of ketamine in nonhuman primates (NHPs) (Gil-da-Costa, Stoner, Fung, & Albright, 2013; Javitt, Steinschneider, Schroeder, & Arezzo, 1996; Lakatos et al., 2020), as well as in human volunteers (Musso et al., 2011).

Our long-term goal is to develop an approach that links NMDAR hypofunction to specific constructs at the cellular, circuit, computational, and behavioral level. Studying visual perception in NHPs—and the Chubb illusion, in particular—is well suited for several reasons. First, the visual system of NHPs has been studied in detail and is highly similar to that of humans. This high similarity increases the translational relevance of findings in the animals and can ultimately lead to the development and evaluation of treatment. Second, the behavioral paradigm (Figure 1B) is simple and has successfully been tested on patients with schizophrenia, as well as bipolar disorder (Schallmo et al., 2015). Furthermore, measures of visual surround suppression generalize well across laboratories, and the deficits in schizophrenia have been replicated several times (Dakin et al., 2005; Serrano-Pedraza et al., 2014; Yang, Tadin, Glasser, Hong, Blake, & Park, 2013; Yoon, Rokem, Silver, Minzenberg, Ursu, Ragland, & Carter, 2009). Third, extracellular recordings in animals (Angelucci, Levitt, Walton, Hupé, Bullier, & Lund, 2002; Jones et al., 2001; Kim & Freeman, 2014) and functional imaging in humans (Seymour, Stein, Sanders, Guggenmos, Theophil, & Sterzer, 2013; Zenger-Landolt & Heeger, 2003) demonstrate that neurons in primary visual cortex are prime candidates for the underlying circuitry, establishing a target for mechanistic, invasive studies.

Based on the NMDAR hypofunction hypothesis, we hypothesized that a subanesthetic dose of ketamine would reduce susceptibility to the Chubb illusion and result in more veridical perceptual reports of contrast, similar to what has been reported in patients with schizophrenia.

## Methods

All procedures were approved by the Rutgers University animal care and use committee and were in agreement with the National Institutes of Health guidelines for the humane care and use of laboratory animals and the ARVO Statement for the Use of Animals in Ophthalmic and Vision Research.

### Animals

Two adult male rhesus macaques (*Macaca mulatta*) performed a visual discrimination task for liquid reward while seated in a standard primate chair, head free, at a distance of 57 cm from a cathode-ray tube monitor (refresh rate, 150 Hz or 120 Hz). An infrared eye tracker (EyeLink 1000; SR Research, Ottawa, Ontario, Canada) recorded their eye position at a sampling rate of 1000 Hz.

### Visual stimuli

We used sinusoidal gratings with a circular aperture, a spatial frequency of two cycles per degree of visual angle (dva), and a diameter of 3 dva; one of the gratings was located 5 dva to the left and the other 5 dva to the right of the center of the screen (Figure 1A). The grating pattern inside the circular aperture (center, C) moved rightward with a speed of 4 dva per second. One grating (“reference”) was always at 25% Michelson contrast, and the other grating (“test”) had a contrast that varied across trials: 5%, 10%, 15%, 20%, 25%, 32.5%, 40%, 47.5%, or 55%. On some trials, one or both gratings were presented with a surround (S), which resulted in three conditions, center versus center (CvC; Figure 1A1), center versus center + surround (CvCS; Figure 1A3) and center + surround versus center + surround (CSvCS; Figure 1A2). The surround annulus matched all parameters of the center grating, except for its diameter (6 dva) and contrast (60%). In two sessions of one of the monkeys, the contrast of the surround was 65%. Background luminance was constant at 30 cd/m^2^. All patterns (C or CS) were equally likely to appear on the left or the right side of the screen.

### Experimental design

Immediately after the monkey fixated a small red dot at the center of the screen within a 4 × 4 dva window, the grating stimuli appeared for 1000 ms, followed by two red dots at the location of the center gratings (Figure 1B). The monkeys had 2000 ms to make a saccade to either dot, indicating which of the center gratings had the higher contrast. Each trial was followed by a 1000-ms gray screen intertrial interval (Figure 1B), during which the animal could receive a liquid reward. If central fixation was lost during the presentation of the gratings or if no saccade was made within 2000 ms, the trial was aborted and not used in the analysis. Trials were grouped in blocks of 21 pseudorandomly interleaved conditions (below).

Training the animals to do this task is complicated by the fact that the animals are motivated to receive reward but not to report what they see honestly. This implies that the condition in which we expect a strong Chubb illusion (CvCS, with similar test and reference contrast) must be rewarded at random (i.e., irrespective of the animal's response); otherwise, they will eventually report whatever was rewarded. However, providing many random rewards typically leads to random responses (presumably, because the animal keeps searching, in vain, for a response that leads to more reward). We solved this here as we did in previous studies (Klingenhoefer & Krekelberg, 2017; Krekelberg & Albright, 2005; Krekelberg, Dannenberg, Hoffmann, Bremmer, & Ross, 2003) by not measuring the full psychometric curve in the condition that results in the illusion (CvCS) but instead focusing on three critical points on the curve: the far left (test contrast 5%), middle (test contrast 25%), and far right (test contrast 55%). These three points are insufficient to fully constrain the parameters of a psychometric curve, but, as detailed in the data analysis section, they suffice to quantify the animals’ susceptibility to the illusion while also allowing us to control for generalized deficits in performance.

In a block of 21 trials, each condition was presented once in pseudorandom order: CvC and CSvCS with eight test contrasts (16 trials), CvCS with 5% or 55% test contrast (two trials), and CvC, CSvCS, and CvCS with test contrast matching the reference (three trials). In the three conditions (CvCS, CvC, and CSvCS) where the test and reference contrast were both 25%, we gave the reward randomly. In all other conditions, a reward was given for a veridical report. Note that this includes the 5% and 55% test CvCS conditions. Essentially, we assumed that the illusion could not be so large as to make a 55% test appear of lesser contrast than a 25% reference. Studies in humans and our own data show that this assumption is warranted (Chubb, Sperling, & Solomon, 1989; Schallmo et al., 2015).

### Training

Training took place over several months. We first trained the animals on the CvC conditions with the (easy) test contrasts of 5% and 55%. Once they were able to chose the correct stimulus with near-perfect accuracy, we introduced the more difficult test contrast (i.e., 10% and 47.5%) and proceeded to the next pair of contrasts only after the animal achieved stable performance on those tests. When we had obtained reliable psychometric curves for the CvC condition, we introduced the CSvCS condition and repeated the training procedure, starting again with the easiest contrasts (5% and 55%).

We introduced the critical CvCS condition only after we obtained reliable and robust psychometric curves for (randomly interleaved) CvC and CSvCS conditions. The CvCS training also initially used only test contrasts of 5% and 55% (rewarded for veridical report). After performance had stabilized, we introduced the critical 25% test contrast (rewarded randomly; see above). Experiments with injections of ketamine or saline started after performance had stabilized on all conditions in the full paradigm.

### Pharmacological manipulation

The animals received an intramuscular injection of 0.4 mL saline during six sessions and 0.3 mg/kg of ketamine added to or diluted in 0.4 mL saline in six other sessions. The monkeys received the injections in their home cage and started the experiment on average within 10 minutes after the injection. Saline and ketamine sessions were always on 2 consecutive days; the order was counterbalanced. Repeated session pairs were separated by at least 72 hours (average 436 hours) to prevent the development of ketamine tolerance. Only data recorded during these 24 sessions were analyzed quantitatively and are reported here.

### Eye movements

We also tested whether ketamine induced changes in fixation accuracy and stability and whether these changes were related to changes in perception. We defined fixation accuracy as the distance (in dva) between the eye and the fixation target during stimulus presentation and fixation instability as the distance the monkey's gaze traveled during stimulus presentation. Specifically, we determined the distance between successive samples of the eye position during stimulus presentation, discarded differences below the nominal resolution of the eye tracker (0.1 dva), and summed the remainder per trial. Given that the nominal resolution underestimates the true resolution, this is likely an upper bound on the total displacement of the eye during the trial. This measure of fixation instability combines displacement across eye movement types (e.g., drift, microsaccades), which are difficult to disentangle in head free recordings.

To assess the relation between changes in fixation and changes in the Chubb illusion, we used a logistic regression (fitglme function in MATLAB, distribution type was “binomial,” and the link function was “logit”). In this regression, the dependent variable was “choose test” (CT; whether the animal chose the test on a given trial), and drug (ketamine/saline), fixation accuracy, and fixation instability were the independent variables.

### Data analysis

We used the MATLAB function pfit set to 4999 runs (Wichmann & Hill, 2001) to estimate psychometric curves as cumulative Gaussians for the CvC and CSvCS stimulus conditions with the lapse rates (gamma and lambda) at the lowest and highest contrasts constrained (independently) to be between 0 and 0.15. The lapse rate, the point of subjective equivalence (parameter), and the slope (parameter) served to quantify each animal's performance on the contrast discrimination task. We used the explained variance as a measure of the goodness of fit for the psychometric curves.

In human subject studies, a change in the point of subjective equivalence (PSE) in the CvCS typically serves to quantify the strength of the illusion. However, as explained above, in animals working for liquid reward an estimate of the psychometric curve (and therefore the PSE) is not feasible in the CvCS condition. Therefore, we used the CvCS condition in which the test contrast equaled the reference (25%) to quantify the strength of the Chubb illusion. In this condition, choosing the test stimulus on 50% of trials (CT = 50%) corresponded to veridical contrast perception and hence the absence of a Chubb illusion. Healthy human observers typically chose the test stimulus on less than 50% of trials (CT ∼ 25%, as in Schallmo et al., 2015). We quantified the ketamine effect (KE) as the difference between CT on days with a ketamine injection and CT on days with a saline injection. If ketamine had no influence, then, on average, KE would be 0. If ketamine increased the strength of the Chubb illusion, then KE would be negative; if ketamine decreased the illusion, then KE would be positive. Based on the NMDAR hypofunction theory, we predicted a positive KE.

To test whether the monkeys’ overall performance suffered due to ketamine (i.e., reduced accuracy due to attentional lapses or general impairment after ketamine injections), we quantified performance (percent correct) for the two easiest test contrasts (Figure 3A). To provide a direct comparison of the change in the illusion strength (i.e., KE) with any overall change in performance, we subtracted the ketamine-induced change in performance (percent correct on the easiest test contrasts) from KE. We refer to this measure as illusion-versus-performance (IvP). If ketamine affects contextual integration specifically, then IvP should be positive. If ketamine merely affects overall performance, then IvP values should be near zero (or negative).

In each of our measures of interest (KE, percent correct, IvP, fixation accuracy, and stability) we compared a group of saline and ketamine trials. To avoid parametric assumptions about the underlying distributions, we used a bootstrap permutation test to assess statistical significance. We randomly assigned trials to either the saline or the ketamine condition, calculated the relevant measure from these relabeled trials, and repeated this 10,000 times to generate a distribution of expected values under the null hypothesis (null distribution). During each iteration, we selected 80% of each group's trials, without replacement. Each relabeled group contained the same number of trials, half from the saline condition. We defined a significant true effect as one that was larger than the 95th percentile of this null distribution (corresponding to a one-tailed test with a significance level of 0.05). Analogous to the Cohen's *d* effect size measure, we quantified the KE effect size as the ratio of the observed, average KE and the standard deviation of the KE null distribution.

## Results

Figure 1A3 illustrates the Chubb illusion. A grating surrounded by a high-contrast annulus appears to have a lower contrast than an identical grating without a surround. The animals were trained to indicate which of two central gratings on the screen appeared to have the higher contrast.

### Contrast discrimination performance

**Figure 2.**
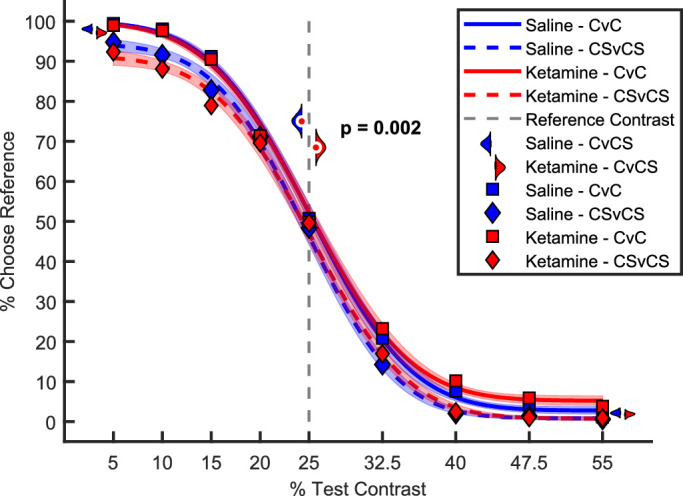
NMDAR hypofunction reduces the Chubb illusion. Because the results were highly similar, and for visual clarity, this figure shows data pooled across animals. Animals indicated which of two gratings had the higher contrast—the test grating with the contrast shown along the horizontal axis or the reference grating with a constant (25%) contrast. Psychometric curves for performance are shown separately for trials without surround annulus (CvC; solid curves) and trials with annuli surrounding both reference and test (CSvCS; dashed curves). Squares show the average choice at each test contrast for the CvC condition, and diamonds for the CSvCS condition. Trials in which only the test stimulus was surrounded by an annulus (CvCS) are presented as semi-violin plots. Colors are used to indicate data obtained after saline (blue) or ketamine (red) injections. Together, these data show that the animals were susceptible to the Chubb illusion and that ketamine specifically reduced the strength of the illusion, without an overall, non-specific change in task performance.

The blue elements in Figure 2 show performance after saline injection (control). We first quantified the animals’ contrast discrimination performance using conditions in which either both (CSvCS) or none (CvC) of the gratings were surrounded by a high contrast annulus. The psychometric curves (Figure 2) were excellent fits to the data (>99% explained variance for all curves). The animals performed very well on the CvC and CSvCS tasks. First, they had no appreciable bias, as evidenced by the close match between the PSE of the psychometric curve (CvC, 25.2%; CSvCS, 24.7%) and the true contrast of the reference (25%). Statistically, the 95% confidence intervals (CIs) of the PSE overlapped with 25% contrast in all conditions and for both animals. Second, the animals’ sensitivity (the slope of the psychometric curve at the PSE) was high (CvC, 8.1; CSvCS, 7.5), and performance for large contrast differences was nearly perfect (lapse rates < 5.7%).

### Surround suppression

In the critical condition leading to the Chubb illusion in healthy human subjects, the animals were asked to pick the higher contrast from two identical gratings—one with a surrounding annulus (“test”) and one without (“reference”) (Figure 1A3). The blue half-violin plot at 25% test contrast in Figure 2 shows that the animals were approximately fourfold more likely to indicate that the reference, the grating without a surround, had the higher contrast. This shows that the animals were indeed susceptible to the Chubb illusion; they picked the test in only 25.0% of trials (CI, 22%–28%). This value falls in the range reported for healthy human subjects (Schallmo et al., 2015).

### Reduced surround suppression due to ketamine

After establishing susceptibility to the Chubb illusion in the NHPs, we proceeded to test our main hypothesis that NMDAR hypofunction reduces the strength of the Chubb illusion. The red elements in Figure 2 show the results on ketamine days. In the critical CvCS condition at 25% test contrast, the animals picked the test in 31.6% of trials (CI, 28.4%–34.8%). As can be inferred from the distributions shown as semi-violin plots in Figure 2, this difference of 6.6% was statistically highly significant (*p* = 0.002, one-tailed permutation test) and corresponds to a large effect size (Cohen's *d* = 2.96) demonstrating that an injection of ketamine indeed reduced susceptibility to the Chubb illusion. We also analyzed this key finding for each monkey separately and found that the Chubb illusion decreased significantly, and to a similar degree, in both monkeys (monkey 1, 8.2%, *p* = 0.003; monkey 2, 5.2%, *p* = 0.022, one-tailed permutation test).

### Non-specific performance changes due to ketamine

**Figure 3.**
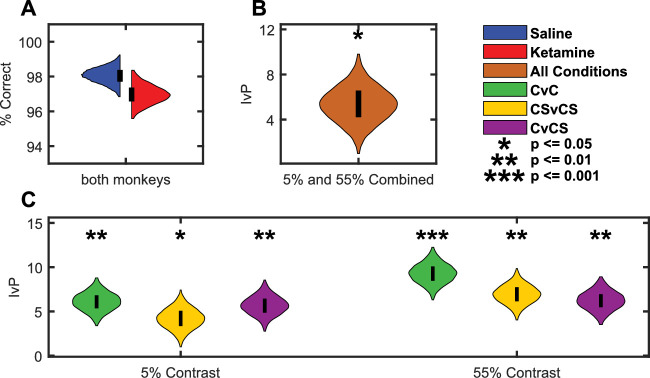
NMDAR hypofunction does not alter performance. (**A**) Near-perfect performance at the easiest contrasts (5% and 55%), regardless of injection type. (**B**) The violin plot shows the distribution of IvP—that is, the ketamine-induced change in the illusion (only CvCS condition) compared with the ketamine-induced change in percent (%) correct at the easiest contrast conditions (5% and 55%) averaged across all tasks. (**C**) Each violin plot shows the distribution of IvP separately for each task (colors) and easy test contrast (*x*-axis). These control analyses show that ketamine changed the perceived strength of the Chubb illusion more than it changed performance at the easiest contrasts. This argues strongly that the low dose of ketamine interfered specifically with contextual integration and did not induce major drowsiness

Because ketamine (at higher doses) acts as a dissociative anesthetic, there is a concern that non-specific changes in behavior (e.g., an overall tendency to poor performance, drowsiness) could be responsible for the reduced strength of the illusion. Comparison of the red and blue psychometric curves in Figure 2, however, shows that ketamine did not substantially affect overall performance on the CvC and CSvCS contrast discrimination tasks. To quantify this, we compared the animals’ performance at the easiest contrasts (in CvC, CSvCS, and CvCS tasks). The overlapping percent correct distributions (Figure 3A) show that ketamine affected this performance only minimally (performance was reduced by only 1.1% on average; p = 0.28, permutation test). This suggests that the change in the illusion strength cannot be explained by a non-specific drop in performance caused by ketamine.

However, this conclusion relies on a null result (that there is no significant change in overall performance due to ketamine). As an additional control analysis and to further strengthen our conclusion, we also compared the ketamine-induced change in the illusion (KE, defined in terms of a percentage of trials; see Methods) with the ketamine-induced change in performance for the easiest test contrasts (percent correct, also defined as a percentage of trials). Figure 3B shows the distribution of this measure (IvP; see Methods) for all tasks combined. The panel shows that ketamine changed the illusion strength significantly more than it changed the performance on the easy tests (IvP = 5.5%; CI, 3.0%–8.1%; *p* = 0.011, one-tailed permutation test). Furthermore, in Figure 3 we calculated IvP separately for each condition and for each of the two easy test contrasts. In all comparisons, ketamine affected the illusion strength more than the overall performance (mean IvP, 4.2–9.4; *p* ≤ 0.049, one-tailed permutation test).

### Eye movements

The head-free recording, together with the large fixation window (see Methods), in principle allowed for large deviations between eye position and the fixation dot. Contrary to this expectation, we found that the animals’ fixation accuracy (mean distance from the fixation target) was excellent, regardless of injection type (saline: mean = 0.67 dva, CI, 0.67–0.68; ketamine: mean = 0.73 dva, CI, 0.73–0.74). The difference between the ketamine and saline trials was small but statistically significant (mean difference [M] = 0.06 dva; CI, 0.05–0.07; *p* ≤ 0.001, permutation test). In other words, ketamine reduced fixation accuracy. Fixation instability (the total distance traveled during stimulus presentation; see Methods) was low as well (saline: mean = 1.46 dva; CI, 1.43–1.49; ketamine: mean = 1.17 dva; CI, 1.15–1.2), and lower during ketamine (M = –0.28 dva; CI, –0.32 to –0.24; *p* ≤ 0.001, permutation test).

Next, we investigated whether eye movements affected the animal's perceptual choices in the illusory CvCS condition. A logistic regression (see Methods) showed that fixation accuracy did affect the animals’ choices, β = 0.65, *SE* = 0.18, *t*(1879) = 3.68, *p* < 0.001; however, fixation instability did not, β = –0.03, *SE* = 0.03, *t*(1879) = –0.81, *p* = 0.42. In other words, the animals chose the test grating with surround significantly more often in trials with lower fixation accuracy; the illusion was weaker in trials with lower fixation accuracy. Importantly, even after accounting for this influence of fixation accuracy, ketamine was still a strong predictor for the animals’ choices, β = 0.27, *SE* = 0.1, *t*(1879) = 2.5, *p* = 0.011. This shows that eye movements may have modulated the illusion strength, but ketamine also reduced the illusion in an eye-movement–independent manner.

## Discussion

We demonstrated that healthy NHPs—just like healthy human volunteers—report a robust Chubb illusion. This provides further evidence that the human and nonhuman primate visual systems are highly similar, not only in terms of their structure and function (Orban, Van Essen, & Vanduffel, 2004) but also in terms of their susceptibility to visual illusions (Klingenhoefer & Krekelberg, 2017; Krekelberg & Albright, 2005; Krekelberg et al., 2003; Subramaniyan, Ecker, Berens, & Tolias, 2013). Our main finding is that a subanesthetic dose of ketamine reduced the Chubb illusion in NHPs. At the low doses used here, ketamine primarily affects the NMDA receptor (Zanos et al., 2018); hence, this provides experimental support for the hypothesis that NMDAR hypofunction causes highly specific impairments in contextual integration, similar to those observed in schizophrenia.

Studying behavioral reports in a patient population often suffers from the problem of generalized performance deficits. Put colloquially, a patient may perform worse on a task simply by “not feeling well,” which increases distraction or reduces motivation. A major advantage of the task used here is that the performance of patients with Sz cannot be explained in such terms. In fact, patients perform better on the task, as they report contrast more veridically than healthy controls (Dakin et al., 2005). The same was true for our NHP subjects; ketamine did not introduce an overall performance deficit, but a very specific improvement in contrast discrimination performance (quantified by a reduction of the Chubb illusion). Such specificity is advantageous both for diagnostic purposes (Silverstein et al., 2013) and to link behavioral changes to underlying neural circuitry.

Because eye movements reduce effective contrast through passive gray-out and active suppression (Ibbotson & Krekelberg, 2011), they could, in principle, reduce the effective contrast of the surround annulus and thereby reduce the illusion strength. (Any influence on the test and reference center gratings would be equal and cancel out.) Our data show that, even after accounting for the correlation between eye movements and the perceptual decision, ketamine reduced the illusion. We note, however, that the evidence for the involvement of eye movements is correlational; given the overall accuracy and stability of fixation, we believe that the causal contribution of eye movements to the illusion is probably modest at best. In our opinion, it seems more likely that eye movement and perceptual abnormalities result largely independently from NMDAR hypofunction in, respectively, the oculomotor and visual systems (e.g., due to local variations in ketamine concentrations). Future experiments with local (sub)cortical application of NMDAR antagonists may be able to reveal the full chain of causation.

Eye movement abnormalities are indicators of neuropsychiatric disease. For Sz specifically, a compound measure quantifying smooth pursuit, saccade, and fixation can classify patients and controls with sensitivity of 78% and selectivity of 94% (Miura et al., 2014). Our findings and earlier studies of reflexive eye movements evoked under ketamine anesthesia (Leopold, Plettenberg, & Logothetis, 2002) suggest that these abnormalities may have an origin in NMDAR hypofunction. Future work could test this hypothesis by probing the influence of low-dose ketamine injections on a wider range of eye movement tasks.

Electrophysiological studies have established that a surround reduces the neural response to a center stimulus. This likely neural correlate of the Chubb illusion relies critically on NMDAR-dependent (Self et al., 2012), strong recurrent connections, including excitation and inhibition within primary visual cortex but also feedback from higher areas (Angelucci et al., 2002; Nurminen & Angelucci, 2014; Shushruth, Nurminen, Bijanzadeh, Ichida, Vanni, & Angelucci, 2013). These recurrent connections form a versatile circuit (del Mar Quiroga, Morris, & Krekelberg, 2016; del Mar Quiroga, Morris, & Krekelberg, 2019; Joukes, Yu, Victor, & Krekelberg, 2017) that performs contextual integration of low-level features (as seen in the Chubb illusion) but also higher level, cognitive features such as the distinction between figure and ground (Self et al., 2012; Van Kerkoerle, Self, Dagnino, Gariel-Mathis, Poort, Van Der Togt, & Roelfsema, 2014) and the allocation of attentional resources (Reynolds & Heeger, 2009). Our findings are consistent with the view that NMDAR hypofunction impairs this circuit, and, considering the versatility of the circuit, this can have numerous behavioral consequences. Current models of this circuitry are sophisticated (Ozeki, Finn, Schaffer, Miller, & Ferster, 2009; Schwabe, Ichida, Shushruth, Mangapathy, & Angelucci, 2010) but not yet at the level of cellular detail necessary to make specific, quantitative predictions. Nevertheless, we can speculate that, beyond the Chubb illusion, other aspects of impaired contextual integration in schizophrenia (Keane et al., 2014; Keane, Paterno, Kastner, Krekelberg, & Silverstein, 2019; Silverstein & Keane, 2011; Uhlhaas, Linden, Singer, Haenschel, Lindner, Maurer, & Rodriguez, 2006; Uhlhaas, Phillips, Mitchell, & Silverstein, 2006) may also be attributable to this canonical circuit. Beyond visual cortex, similar microcircuits exist in other sensory, as well as cognitive control areas (Phillips & Silverstein, 2013), which leads to the prediction that NMDAR hypofunction could also contribute to cognitive symptoms observed in schizophrenia.

Ketamine is actively investigated as a treatment for chronic pain, as well as depression, but potential side-effects, especially those of repeated administration, are poorly monitored in the majority of studies (Short, Fong, Galvez, Shelker, & Loo, 2018). In light of the recent approval of a nasal ketamine spray as a treatment for depression (Food & Drug Administration, 2019), the need for a standardized assessment of side-effects becomes particularly pressing. Our findings predict specific alterations of visual perception in ketamine users, and they show that such alterations can be quantified reliably. We therefore suggest that standardized assessments of ketamine side-effects should include simple tasks, such as the one used here, that depend on the NMDA receptor. Such assessments could generate insight into the perceptual and cognitive consequences of ketamine use.

## Conclusions

Our study shows that a specific change in visual perception seen in schizophrenia can be reproduced in an animal model with a low dose of ketamine. This provides support for the NMDAR hypofunction theory and builds a foundation on which to study the underlying processes and neural circuitry that may be affected in schizophrenia. Such an approach will benefit from the broad knowledge base and strong quantitative techniques available in the visual system and will have substantial translational relevance due to the extensive structural and functional similarities between the macaque and human visual systems.
